# Acetate supplementation reduces microglia activation and brain interleukin-1β levels in a rat model of Lyme neuroborreliosis

**DOI:** 10.1186/1742-2094-9-249

**Published:** 2012-11-07

**Authors:** Catherine A Brissette, Heidi M Houdek, Angela M Floden, Thad A Rosenberger

**Affiliations:** 1 Department of Pharmacology, Physiology and Therapeutics, University of North Dakota School of Medicine and Health Sciences, 501 North Columbia Road, Grand Forks, North Dakota, 58203, USA; 2 Department of Microbiology and Immunology, University of North Dakota School of Medicine and Health Sciences, 501 North Columbia Road, Grand Forks, North Dakota, 58203, USA

**Keywords:** Neuroborreliosis, Microglia, Astrocytes, Neuroinflammation, Acetate

## Abstract

**Background:**

We have found that acetate supplementation significantly reduces neuroglia activation and pro-inflammatory cytokine release in a rat model of neuroinflammation induced with lipopolysaccharide. To test if the anti-inflammatory effect of acetate supplementation is specific to a TLR4-mediated injury, we measured markers of neuroglia activation in rats subjected to *B. burgdorferi*-induced neuroborreliosis that is mediated in large part by a TLR2-type mechanism.

**Methods:**

In this study, rats were subjected to Lyme neuroborreliosis following an intravenous infusion of *B. burgdorferi* (B31-MI-16). Acetate supplementation was induced using glyceryl triacetate (6g/kg) by oral gavage. Immunohistochemistry, qPCR, and western blot analyses were used to measure bacterial invasion into the brain, neuroglial activation, and brain and circulating levels of interleukin 1β. Statistical analysis was performed using one-way analysis of variance (ANOVA) followed by a Tukey’s post hoc tests or using a Student’s *t* test assuming unequal variances when appropriate.

**Results:**

We found that acetate supplementation significantly reduced microglia activation by 2-fold as determined by immunohistochemical and western blot analysis. Further, acetate supplementation also reduced the expression of the pro-inflammatory cytokine IL-1β by 2-fold as compared to controls. On the other hand, the inoculation of rats with *B. burgdorferi* had no effect on astroglial activation as determined by immunocytochemistry and western blot analysis despite significant increases in circulation levels of antigen toward *B. burgdorferi* and presence of the bacteria in the central nervous system.

**Conclusions:**

These results suggest that microglial activation is an essential component to neuroborreliosis and that acetate supplementation may be an effective treatment to reduce injury phenotype and possibly injury progression in Lyme neuroborreliosis.

## Background

Lyme disease, caused by the spirochete *Borrelia burgdorferi*, is an National Institue of Allergy and Infectious Diseases/ National Institutes of Health Group I priority emerging/re-emerging infectious disease and the most frequently reported arthropod-borne disease of humans in the United States [1]. *B. burgdorferi* can infect immune-competent humans and other vertebrates for extensive periods of time, even for the host’s lifetime [2-4]. The Lyme disease spirochete is an extracellular organism with an affinity for the central nervous system and invades via hematogenous spread [5] and can be isolated from cerebrospinal fluid (CSF) as early as 18 days after the bite from an infected tick [6]. Lyme neuroborreliosis, which occurs in 10 to 15% of untreated patients [7], results in meningitis, headache and facial nerve palsy [5]. *B. burgdorferi* adheres to primary neural cells from mice and rats, as well as glial cell lines, resulting in direct cytotoxicity, increasing brain levels of activated CD8 T cells and B cells [5-7]. Since *B. burgdorferi* does not produce exotoxins, neurological damage is most likely a result of the host’s own inflammatory response, in large part by a TLR-2-mediated cellular recognition [8] that increases levels of pro-inflammatory cytokine IL-1β, IL-6, IL-8, TNF-α, and CXCL13 in the CSF [9].

Dietary acetate is a potentially effective therapy for the treatment of Canavan disease [10] and reduces neuroinflammatory phenotype in rats subjected to neuroinflammation [11]. In brain, acetate is converted to acetyl coenzyme A (acetyl-CoA) through the combined action of nuclear acetyl-CoA synthetase 1 [12] and mitochondrial acetyl-CoA synthetase 2 [13]. When acetate is supplied by a single oral dose of glyceryl triacetate (GTA), brain acetyl-CoA levels increase by 2.2-fold, it reduces neuroglia activation by 40 to 50% [11], increases histone acetylation [14], and is anti-inflammatory with regard to reducing IL-1β in a rat model of neuroinflammation [15]. Further, in cultured microglia, acetate treatment shifts the release of cytokines to a more anti-inflammatory state through mechanisms that involve both histone and non-histone protein acetylation [16]. These data suggest that altering acetyl-CoA metabolism may be active at modulating the neural immune response. In this regard, acetyl-CoA is a widely active precursor in numerous biological processes that is central to mitochondrial energy supply, fatty acid synthesis, and lipid metabolism [17]. In addition, acetyl-CoA is utilized as a substrate for protein acetylation which, when it occurs on nuclear histones, leads to chromatin architectural changes and changes in gene expression [18]. Therapeutically, increases in the histone acetylation are implicated as being protective in animal models of cerebral ischemia [19], neuroinflammation [11], and amyotrophic lateral sclerosis [20]. An increase in histone acetylation also reduces microglial activation in traumatic brain injury [21], and restores impaired learning and memory in neurodegenerative diseases [22].

The focus of this study was to evaluate acetate supplementation as a therapeutic strategy to reduce neuroinflammation in rats subjected to Lyme neuroborreliosis. Because an increase in acetyl-CoA metabolism is driven by alterations in intracellular acetate utilization, we believe that this therapy can be used to effectively attenuate the TLR2-induced neural immune response as found in Lyme neuroborreliosis. To test this hypothesis, we measured the effectiveness of acetate supplementation to attenuate brain microglial and astroglial activation and decrease brain levels of the pro-inflammatory cytokine IL-1β in rats inoculated with *B. burgdorferi*.

## Methods

### Reagents

Glyceryl triacetate, 2-mercaptoethanol, buffers, fixative solutions, a Cy3-labeled monoclonal anti-glial fibrillary acidic protein (GFAP) antibody (C9205) were purchased from Sigma-Aldrich (St. Louis, MO, USA), and Texas Red™-labeled tomato lectin antibody (TL-1176), normal horse serum, normal goat serum, and Vectashield™ were obtained from Vector Laboratories (Burlingame, CA, USA). An antibody recognizing integrin alpha M chain (CD11b), the rat homolog of the human C3bi complement receptor, a non-labeled GFAP antibody, an alpha tubulin antibody 3,3^′^-diaminobenzidine tetrahydrochloride, and a biotinylated goat anti-rabbit immunoglobulin G (IgG) were purchased from Millipore (Billerica, MA, USA) and a fluorescein-conjugated anti-*B. burgdorferi* polyclonal antibody and isotype control antibody was purchased from KPL (Gaithersburg, MD, USA). Absolute ethanol was from Pharmco (Brookfield, CT, USA) and all other reagents unless noted otherwise were purchased from EMD Chemicals (Gibbstown, NJ, USA).

### Bacterial culture

Virulent *B. burgdorferi* strain B31-MI-16 [23,24] was grown at 34°C to cell densities of approximately 1 × 10^7^ mL in modified Barbour-Stoenner-Kelly II (BSK-II) medium [25]. Bacteria were pelleted by centrifugation (6000 × g, 10 min) and washed three times with phosphate-buffered saline (PBS; 9.6 mM NaH_2_PO_4_, 0.73 mM KH_2_PO_4_, 137 mM NaCl, 2.7 mM KCl, pH 7.4). Bacteria were enumerated by dark field microscopy using a Petroff-Hausser chamber (Hausser Scientific, Horsham, PA, USA).

### Animal studies

This study was conducted in accordance with the NIH Guidelines for the Care and Use of Laboratory Animals under an approved by the University of North Dakota Institutional Animal Care and Use committee using male Sprague–Dawley rats (Charles River, Wilmington, MA, USA). All rats were acclimated in our facility for at least seven days prior to inclusion in the study and maintained on a constant 12 h light cycle. Rats were fed a standard 2018 Teklad Global 18% protein rodent diet (Harlan, Madison, WI, USA) *ab libidum*. Infected rats were inoculated with *B. burgdorferi* bacteria via an intravenous tail vein injection at a dose of 5 × 10^5^ bacteria per rat. Following inoculation, rats were treated daily with either GTA or water (6 g/kg body weight) by oral gavage. At 28 days the rats were anesthetized with an intravenous injection of pentobarbital (50 mg/kg) and those used for western blot analysis were subjected to decapitation, brains were removed, flash frozen in liquid nitrogen, and stored at −80°C until use. Rats used for immunohistochemistry were euthanized by cardiac perfusion with 0.9% saline containing 1000 units/L sodium heparin (Baxter, Deerfield, IL, USA) followed by a mixture of 4% paraformaldehyde and 2% glutaraldehyde in 0.1 M phosphate buffer. Whole brains were removed then post-fixed in the same paraformaldehyde mixture for 48 h at 4°C. The fixed brains were transferred to 0.1 M PBS, pH 7.4 and stored at 4°C.

### Enzyme-linked immunosorbent assay (ELISA)

Rat blood was drawn from the tail artery and collected in heparin-coated tubes. Blood samples were centrifuged (6,000 × g) to remove red blood cells and the serum was stored at −20°C. To measure rat IgM against *B. burgdorferi*, 96-well plates were coated overnight with 10 μg/mL *B. burgdorferi* lysate (mid-log phase *B. burgdorferi* B31-MI-16) in carbonate coating buffer (0.32g Na_2_CO_3_, 0.586g NaHCO_3_ per 200 mL, pH 9.6) at 4°C. Protein concentration was determined using a bicinchroninic acid assay (Pierce, Rockford, IL, USA). Room temperature plates were washed once with PBS containing 0.05% Tween 20 (by Vol., PBS-T). Wells were blocked for 2 h at room temperature with PBS containing 2% bovine serum albumin (BSA) then washed three times with PBS-T. At the time of the assay a 1:100 dilution of serum in PBS was placed on the plate and incubated for 2 h at 37°C. Wells were washed three times with PBS-T then incubated for 1 h at room temperature with horseradish peroxidase-conjugated goat antiserum against rat IgM diluted to 1:5000 in PBS, (AbD Serotec, Raleigh, NC, USA). Color development was performed using a tetramethylbenzidine substrate (TMB; Thermo Scientific, Rockford, IL, USA) for 15 min, and stopped with the addition of 2 N sulfuric acid. Circulating IL-1β levels were measured using ELISA according to the manufacturer’s instructions (catalog number RLB00, R&D Systems, Minneapolis, MN, USA). Plates were incubated for 2 h at room temperature, washed using aspiration, followed by the addition of an antibody conjugate then incubation for 2 h at room temperature. The antibody conjugate was washed from the plates and IL-1β was detected by adding a chromogenic substrate. Absorbance from both assays was measured at 450 nm using a BioTek Epoch plate reader with KC4 software (BioTek, Winooski, VT, USA).

### Analysis of *B. burgdorferi* recA DNA levels

Total DNA was extracted from frozen tissue samples using a DNeasy kit (Qiagen, Valencia, CA, USA) as described in the manufacturer’s instructions. A quantitative polymerase chain reaction (qPCR) was performed by using a Bio-Rad MyiQ2 thermal cycler (Bio-Rad, Hercules, CA, USA) where each run included a sample that lacked template to measure DNA contamination. Oligonucleotide primers used for amplification were *B. burgdorferi recA* nTM17F 5^′^GTGGATCTATTGTATTAGATGAGGCTCTCG3^′^; *B. burgdorferi recA* nTM17R 5^′^ GCCAAAGTTCTGCAACATTAACACCTAAAG3^′ ^[26]; and RT^2^ qPCR primer set for rat cyclophilin A (Ppia) (catalog number PPR06504A, SABiosciences, Frederick, MD, USA). The qPCR was performed in 40 cycles following an initial 10 min denaturation at 95°C. Each cycle consisted of a 1 min annealing step performed at 55 or 60°C for *B. burgdorferi* or rat genomic DNA, respectively, followed by a 15 sec melting interval at 95°C. Product melting curves were generated at the end of the reaction using a stepped temperature gradient of 0.5°C × 10 sec starting at 60°C. A 10-fold serial dilution of *B. burgdorferi* (B31-MI-16) genomic DNA or rat genomic DNA was included in every assay for each primer set. This enabled the generation of standard curves from which the amount of DNA present in each sample could be calculated using the Bio-Rad MyiQ2 software. The same software package was also used for melting-curve analyses. To verify amplicon sizes and purities, all products were separated by agarose gel electrophoresis, and DNA was visualized with ethidium bromide. *B. burgdorferi recA* copies were calculated relative to the average triplicate value for the rat cylophilin A housekeeping gene from the same DNA preparation and reported as the averaged values obtained from triplicate runs of each sample.

### Brain protein extraction

Rat brains were removed from storage at −80°C and placed on ice. Each brain was then dissected at the middle carotid artery, and the anterior portion was placed in a scintillation tube with 3 mL of ice-cold extraction buffer containing 50 mM Tris (pH 7.4), 150 mM NaCl, 1 mM EGTA, 1 mM sodium orthovanadate, 5 mM ZnCl_2_, 100 mM NaF, 1 mM PMSF, 0.1% Igepal CA-630, and one Complete™, EDTA-free tablet (Catalog number 05056489001, Roche Applied Science, Indianapolis, IN, USA) per 50 mL [27]. The solution containing the sample was then allowed to sit on ice for 10 min then homogenized using probe sonication until no solid was evident. Once homogenized, samples were centrifuged (4,500 × g) at 4°C for 20 min [28]. The cytosolic extract was aliquoted into 200 μL volumes and stored at −80°C.

### Western blot analysis

Samples were allowed to thaw on ice then prepared for electrophoresis in Laemmli sample buffer containing 5% 2-mercaptoethanol. Electrophoresis was performed on 50 μg of protein using a 15% Tris–HCl gel at 100 volts for 135 min. The protein was transferred onto a 0.45 μm nitrocellulose membrane at 100 volts for 90 min on ice. Primary antibodies (1: 500 dilution) were prepared in 20 mM Tris buffer, pH 7.4 containing 150 mM NaCl, 0.05 % Tween 20 (TTBS), and 5% non-fat dried milk. The nitrocellulose membranes were incubated with primary antibody overnight at 4°C then conjugated with horseradish peroxidase-linked secondary antibody (1:10,000 dilution) for 90 min at room temperature. Protein bands were detected using SuperSignal™ West Femto Chemiluminescent Substrate (Pierce, Rockford, IL, USA) and analyzed in a UVP Bioimaging System (Upland, CA, USA) using LabWorks™ imaging software (version 4.5). All western blot data is expressed as the ratio of the optical density of the target antibody normalized to the optical density α-tubulin and reported as the percent of control. Protein concentrations were measured using the Bradford assay [29].

### Fixation, sectioning, and antigen retrieval

Post-fixed rat brains were equilibrated at 4°C in a 0.1 M phosphate buffer (pH 7.2) solution containing 20% sucrose. Sucrose-impregnated brains were frozen in isopentane (Alfa Aesar, Ward Hill, MA, USA) cooled to −50°C on dry ice, then mounted onto cryostat pedestals with M-1 embedding matrix (Lipshaw, Pittsburgh, PA, USA). A cryostat (IEC, Needham Hts., MA, USA) was used to cut 20 μm serial coronal brain sections, which were mounted on gelatin-coated glass slides. The mounted sections were stored in slide cases at −80°C until use. The sections were rehydrated in 0.1 M Tris-buffered saline, pH 7.6 (TBS) prior to antigen retrieval to enhance antibody-binding efficiency as described [30].

### Immunohistochemistry

Immunohistochemistry was performed using antibody dilutions of 1:1000 for the Texas Red™-labeled tomato lectin, 1:400 for the Cy3-labeled GFAP, and 1:400 for the non-labeled GFAP. White field immunohistochemistry using the non-labeled GFAP antibody with the biotinylated secondary was performed as described [11]. To measure brain bacterial spirochetes, a dilution of 1:5000 was used for the fluorescein-conjugated anti-*B. burgdorferi* antibody and to rule out non-specific binding a fluorescein-conjugated isotype control antibody was used at a dilution of 1:5000. Following antigen retrieval, samples were washed with three 10 min washes in 0.1 M PBS, pH 7.4 + 0.1% Triton™ X-100 (EMD Chemicals, Gibbstown, NJ, USA) (PBS-T buffer). The sections were then incubated for 1 h in blocking solution containing 1% BSA, 0.25% Triton™ X-100, 3% horse serum, and 10% goat serum in PBS then with primary antibody for 24 h at 4°C in hydration chambers. After incubation the sections were washed with PBS, dehydrated with increasing concentrations of ethanol/water solutions (70, 95, and 100% ethanol by Vol.), then equilibrated in xylene/ethanol (1:1, by Vol.) followed by 100% xylene. The dehydrated samples were covered with slips using Vectashield™. Confocal images were collected on a Zeiss LSM 510 META confocal microscope (Carl Zeiss MicroImaging*,* Thornwood*,* NY, USA) (Zeiss AIM software version 4.2 SP1) using a 488 nm Argon laser line, a 543 nm HeNe laser line and appropriate filters for visualizing the fluorophores.

### Statistical analysis

A one-way analysis of variance (ANOVA) followed by a Tukey’s post hoc and assumption tests were used to calculate statistical differences using GraphPad InStat statistical software (version 3.10, GraphPad Software, San Diego, CA, USA). The statistical analyses of brain qPCR data were performed using Student’s *t* test and assuming unequal variances. All results are expressed as means ± SD with significance set at *P* ≤0.05, unless noted otherwise. The sample size of all serum, brain qPCR, and immunohistochemical analyzes were set at six representing sampling from six different rats. Western blot analysis was performed on brain extracts from seven control rats, six rats inoculated with *B. burgdorferi* that were treated with water and five rats inoculated with *B. burgdorferi* and treated with GTA.

## Results

### Induction of neuroborreliosis in the rat

**Figure 1:**
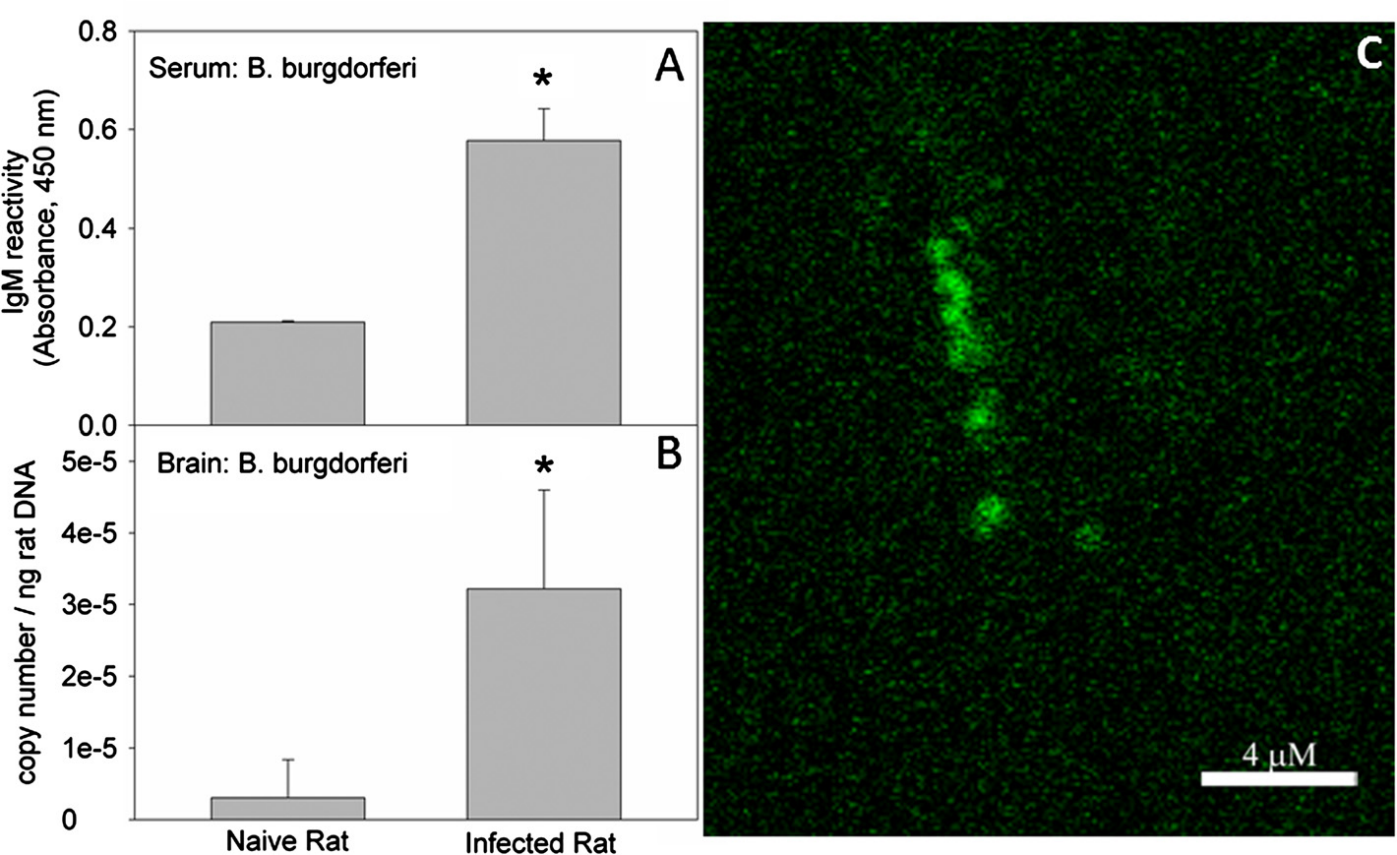
**Induction of neuroborreliosis following a single inoculation with *B. burgdorferi*.** Serum levels of IgM towards *B. burgdorferi* were measured at 28 days post-inoculation in naïve and infected rats, panel (**A**). *recA* copy number was measured to determine the magnitude of brain infection in naïve and infected rats, panel (**B**) and immunohistochemistry was performed to identify the presence of *B. burgdorferi* in an infected rat brain at 28 days post-inoculation, panel (**C**). Values represent the means ± SD of serum immunoreactivity, or brain copy number with a sample size of six representing samples from six different rats per group. The asterisk (*) represents a significant difference (*P* ≤0.05) comparing infected to naïve rats using an unpaired *t* test (GraphPad InStat, version 3.10).

To determine the presence of bacteria in the brain and to gauge the extent by which neuroborreliosis is induced in the rat, we inoculated a cohort of animals with *B. burgdorferi* (5 x 10^5^ cells per rat). At varying times, post-inoculation experiments measuring serum IgM reactivity toward the bacteria, *B. burgdorferi recA* copy number, and immunohistochemistry were performed to identify the presence of the spirochete. These results show that serum *B. burgdorferi* IgM levels at 28 days after the inoculation were 3-fold higher when compared to control naive rats (Figure 1A). These data suggest that the inoculation strategy was effective at increasing circulating levels of *B. burgdorferi* in the blood stream. To assess the presence and the extent by which the bacteria invade the brain we measured *recA* copy number using qPCR focusing on the *recA* gene transcript, a standard marker for the presence of *B. burgdorferi* in which a single copy of *recA i*s present per spirochete. These experiments showed that six out of seven of the infected rats had detectable levels of *B. burgdorferi* in the brain. The content of *recA* transcript was 14-fold above that found in the naïve rat at 28 days (Figure 1B) and increased 2-fold between days 7 and 28 post-infection (data not shown). To further support the presence of bacteria in the brain, an immunohistochemical analysis was performed using a fluorescently labeled antibody toward *B. burgdorferi*. Immunoreactivity toward the *B burgdorferi* antibody was found in all rats inoculated with bacterium (six out of six) showing reactivity in all brain regions analyzed (cortex, hippocampus, and striatum). No immunoreactivity was found in control rat brains (six out of six) and no immunoreactivity was found in inoculated rats screened with a fluorescently labeled isotype control antibody (six out of six). When serial fluorescent images from inoculated rats screened with *B. burgdorferi* antibody were reconstructed into Z-axis stacks, they showed the presence of prototypical *B. burgdorferi* spirochetes (Figure 1C). These data suggest that following an initial inoculation with *B. burgdorferi* at a concentration of 5 × 10^5^ cells per rat, a significant number of bacteria enter the rat brain and possibly continue to reproduce and induce neuroborreliosis.

### Acetate supplementation and neuroglia reactivity in rat subjected to *B. burgdorferi*

**Figure 2:**
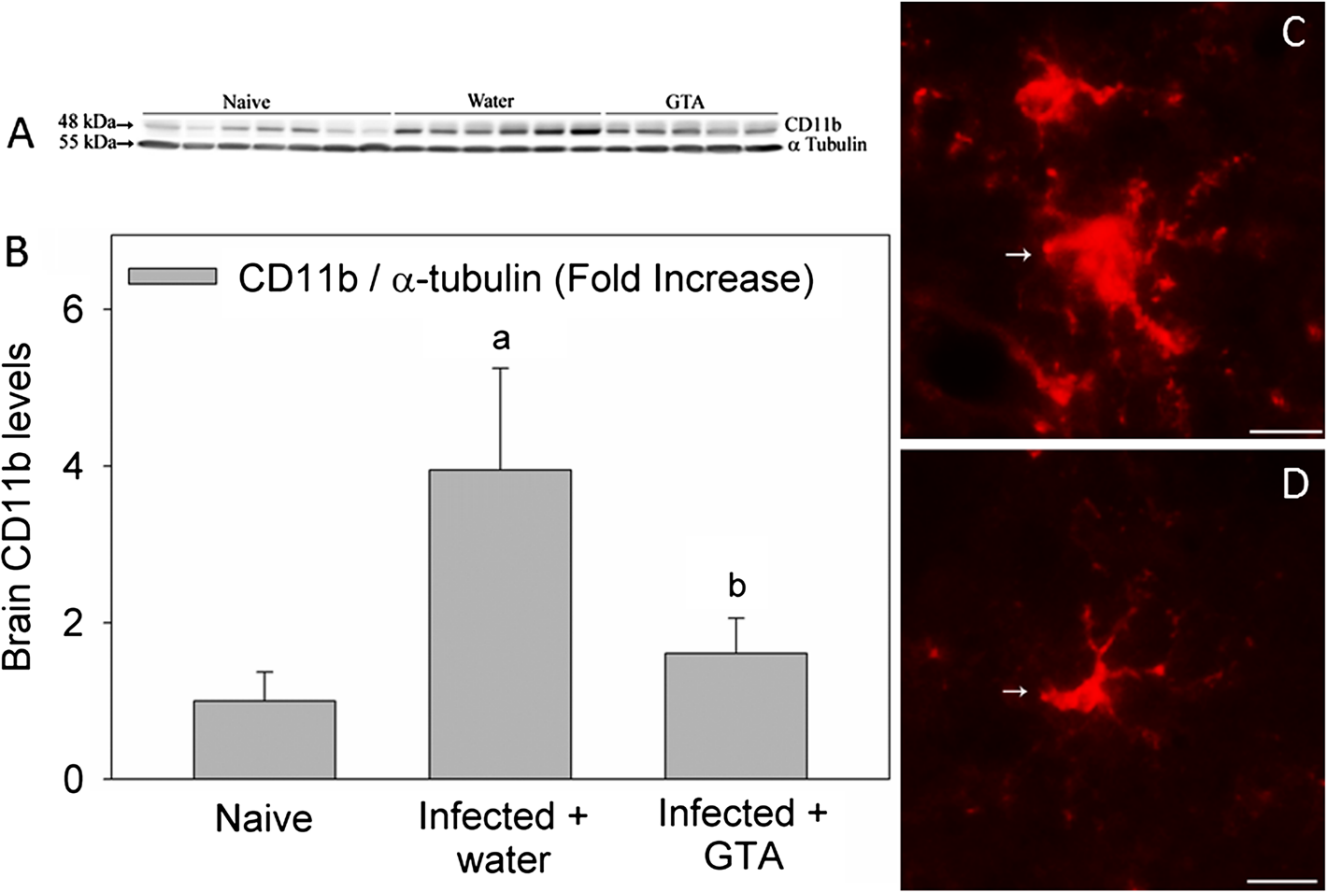
**The effect of acetate supplementation on microglia reactivity in rats subjected to neuroborreliosis**. Panel (**A**) shows a representative image of the western blot analysis measuring integrin alpha M chain (CD11b) and immunoreactivity, a surrogate marker of microglia activation, and panel (**B**) represents the composite measure of the western blot analysis. Panel (**C**) and (**D**) show representative images of tomato lectin-positive microglia (Arrows) in infected rats treated with either water or glyceryl triacetate (GTA), respectively. The values in panel B represent the means ± SD expressed in units of fold-increase over naïve rats where ‘a’ represents a significant increase compared to naïve rats and ‘b’ represents a significant difference comparing infected rats treated with GTA to infected rats treated with water (n = 6, *P* ≤0.05). The reticule in panels C and D represent a length of 10 μm.

**Figure 3:**
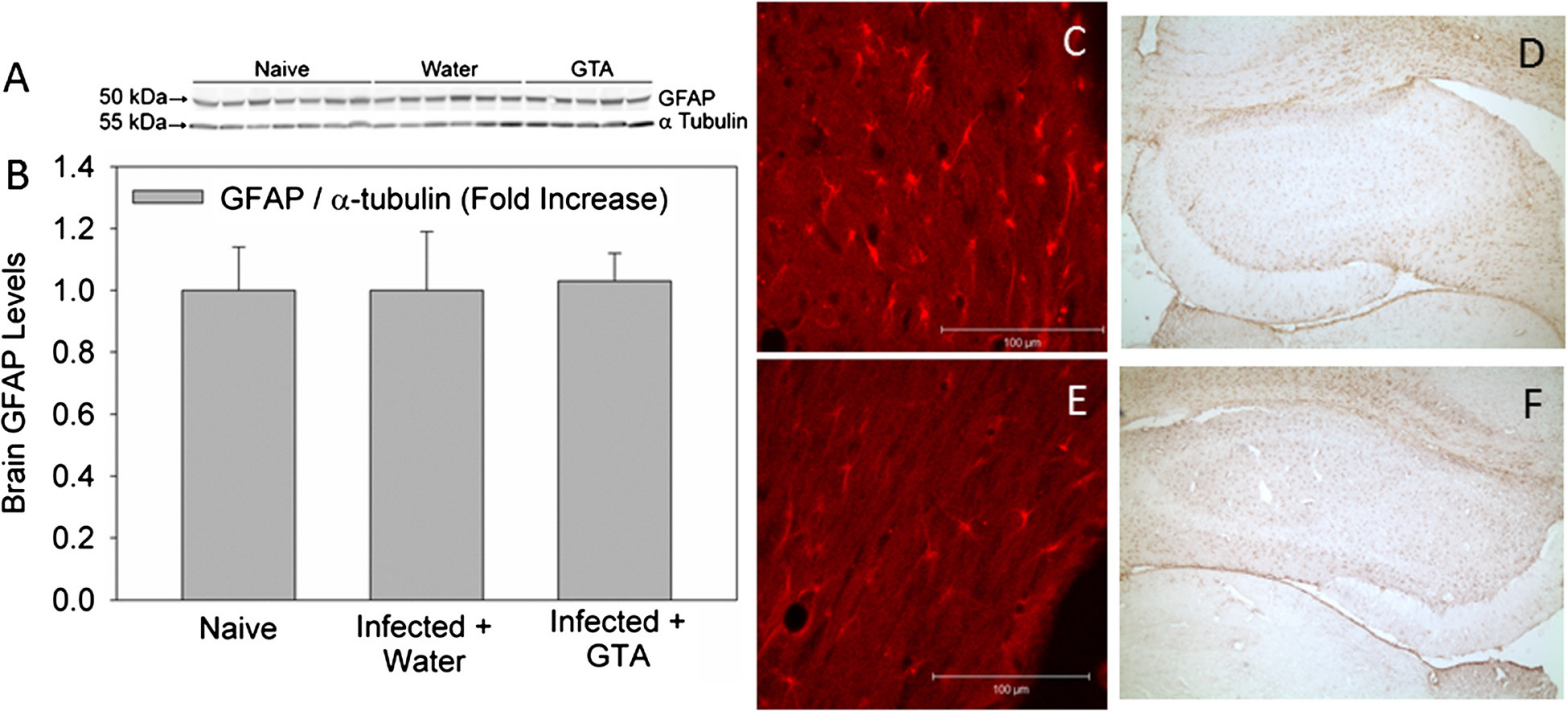
**The effect of acetate supplementation on reactive astrogliosis in rats subjected to neuroborreliosis**. Panel (**A**) shows a representative image of the western blot analysis measuring glial fibrillary acidic protein (GFAP) immunoreactivity, a marker of astroglial activation, and panel (**B**) is the composite measure of the western blot analysis. Panels (**C**) and (**E**) show representative high-magnification images of Cy3-labeled GFAP-positive astroglia in infected rats treated with either water or glyceryl triacetate (GTA), respectively and panels (**D**) and (**F**) show low-magnification images of GFAP-positive astrocytes in infected rats treated with either water or GTA, respectively. The values in panel B represent the means ± SD expressed in units of fold-increase over naïve rats with a sample size of seven (naïve), six (infected + water), and five (infected +GTA).

In an effort to determine the therapeutic efficacy of acetate supplementation on reducing neuroglia reactivity the content of brain CD11b, a marker of microglia activation, and GFAP, a marker of astrocyte reactivity, were performed using western blot analysis on naïve rats and rats subjected to *B. burgdorferi*. To confirm the western blot analysis, immunohistochemical analysis showing morphological changes in lectin-positive microglia and GFAP-positive astrocytes were performed. These experiments show that CD11b protein levels were increased 4-fold in rats subjected to a *B. burgdorferi* infection and treated with water as compared to control naïve rats (Figures 2A and 2B). Conversely, rats that were subjected to *B. burgdorferi* and treated prophylactically with GTA demonstrated a significant reduction in the levels of CD11b to control levels. Immunohistochemical analysis of tomato lectin-positive microglia showed that microglia in water-treated rats display a characteristic ramified structure with an increase cytoplasmic and dendrite volume (Figure 2C). In rats treated prophylactically with GTA, microglia maintain a non-stimulated morphology (Figure 2D) consistent with the western blot analysis. On the other hand, infection did not alter the content of GFAP, the morphology of GFAP-positive astrocytes (Figure 3, C and E), nor did it alter the distribution of GFAP-positive astrocytes (Figure 3, D and F) in the brains of rats treated with either water or GTA (Figure 3 A-D). These data suggest that following infection, one of the primary cells in brain involved in the induction of neuroborreliosis may be the microglia and that *B. burgdorferi* induces, at least in part, the innate immune response in brain. Further, these data suggest that treatment of infected rats with prophylactic GTA can reverse the induction of microglia similar to that found in rats subjected to lipopolysaccharide (LPS)-induced neuroinflammation [11,15].

### Acetate and pro-inflammatory IL-1β levels in rats subjected to *B. burgdorferi*

**Figure 4:**
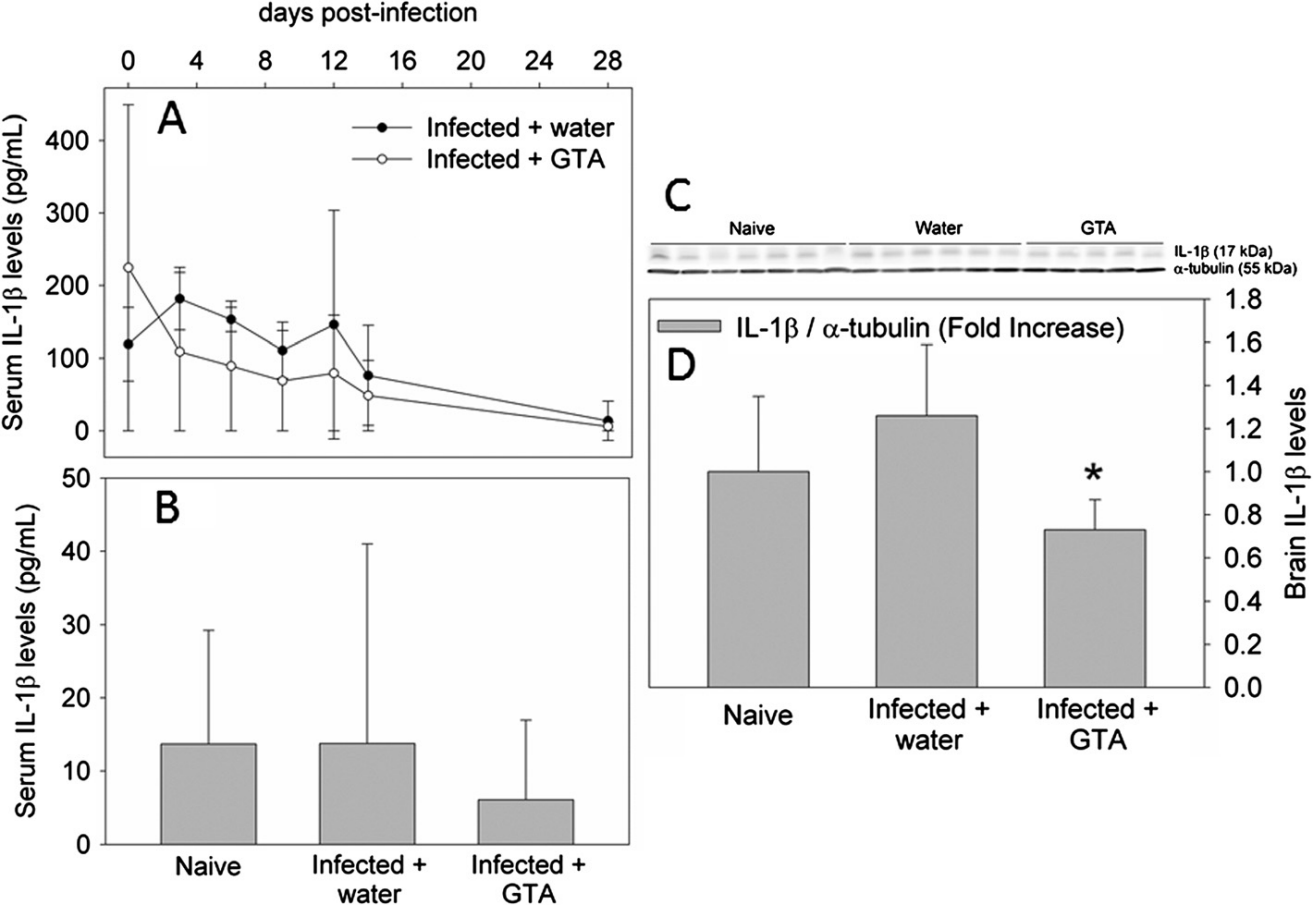
**The effect of acetate supplementation on *B. burgdorferi*-induced increases in brain IL-1β.** Panel (**A**) represents the time-dependent changes in serum IL-1β between the day of inoculation (day 0) and 28 days post-infection showing infected rats treated with either water or glyceryl triacetate (GTA). Panel (**B**) represents serum IL-1β levels at 28 days post-infection in naïve and infected rats treated with either water or GTA. Panel (**C**) shows a representative image of the IL-1β and α tubulin western blots and panel (**D**) represents the composite analysis of the western blot analysis brain IL-1β in naïve and infected rats treated with either water or GTA. Values represent the means ± SD with a sample size of seven, six and five per group, respectively. The asterisk (*) represents a significant difference comparing GTA-treated rats to water-treated rats.

We have demonstrated that one of the functional outcomes of acetate supplementation in attenuating LPS-induced neuroinflammation is a reduction in the expression of the pro-inflammatory cytokine IL-1β [15]. To determine the effect that acetate supplementation has on IL-1β in rats subjected to *B. burgdorferi*, we measured serum changes in this cytokine between the date of inoculation and 28 days and measured brain levels at 28 days. In these experiments, we found that circulating levels of IL-1β did not differ between infected rats treated with either water or GTA at any of the time points measured (Figure 4A). The serum levels of IL-1β in infected rats treated with water, however, were significantly higher at days 3 and 6 compared to day 28 post-infection suggesting that infection does alter circulating levels of this cytokine at least transiently. Serum concentrations on day 28 did not differ between naïve rats and infected rats treated with either water or GTA (Figure 4B). As expected, treatment with GTA significantly reduced brain levels of IL-1β protein by 30% below that found in either naïve or infected rats treated with water (Figure 4C and D). Because circulating levels of IL-1β were not significantly different on day 28 coupled to the reductions in activated microglia suggests that the decrease in IL-1β levels in brain are the result of a reduction in microglia reactivity.

## Discussion

As a proof-of-concept study we measured the effectiveness of acetate supplementation to reverse pro-inflammatory events in the brains of rats subjected to a *B. burgdorferi* infection. In these experiments, we found that the spirochete invaded the rat brain and induced microglial activation. Further, acetate supplementation was effective at reversing brain microglia activation and reduced IL-1β levels, suggesting that treatment may reduce the pathologic sequelae in brain associated with Lyme neuroborreliosis.

Ninety percent of all vector-borne diseases in the Unites States can be attributed to Lyme disease. Because of its importance as an emerging/infectious disease, developing rodent models of Lyme disease is crucial to begin to understand how neuroborreliosis develops, and necessary to develop potential therapeutics to alleviate its long-term debilitating effects on the patient’s health [3,4]. Currently, the primary animal model used to study Lyme neuroborreliosis is the primate rhesus macaque model, in which neuroborreliosis is induced by the intracerebral ventricular injection of *B. burgdorferi*. In this model, there is persistent neuroglial activation, production of high levels of pro-inflammatory cytokine, and neuronal and glial apoptosis [9]. Consistent with these findings we found that following inoculation, rat brain microglia were significantly activated compared to control rats. However, we did not find activation of astrocytes in brains of infected rats as determined by immunohistochemical and western blot analysis. We believe that the lack of reactive astrogliosis found in this study is due in part to our mode of inoculation and the quantity of bacteria introduced. The primate model uses an intrathecal injection into the cisterna magna with 1 × 10^8^ spirochetes [31]. Here, rats were injected with a loading dose of 1 × 10^5^ spirochete given intravenously, which resulted in a much lower challenge. The specifics concerning the development of Lyme neuroborreliosis are unclear, but the role that microglia have in the initiation and progression of this disease is less ambiguous. Microglia are directly activated when exposed to *B. burgdorferi* and involved in neuronal-glia and glial communication [32]. While multiple cell types in the brain express MHC, in studies using minocycline (a suppressor of microglia) microglia activation was essential to induce stress-induced ERK1/2 neuroimmune modulation [33]. Further, the rapid increase in the expression of IL-1 receptor on microglia in response to endotoxin and vascular occlusion support the role of microglia as being a primary initiator of the neuroimmune response [34,35]. Based on this data, we believe an initiating event associated with Lyme neuroborreliosis is the activation of resident microglia. The increase in the number of bacteria found in the brain between 7 and 28 days further supports this premise, although experiments focused on a longer duration will be necessary to determine if microglia activation will stimulate reactive astrogliosis in this model.

At the onset of these studies we proposed that acetate supplementation, through a mechanism involving short chain acetyl-CoA metabolism, would attenuate neuroinflammation induced by *B. burgdorferi* similar to that seen in rats subjected to neuroinflammation [11]. Energy supplementation as a therapeutic strategy to treat neurodegenerative disorders is well appreciated [36-38] and with regard to acetate supplementation works by bolstering acetyl-CoA levels via acetyl-CoA synthetases [12,13]. An increase in acetyl-CoA can stimulate brain energy metabolism, increase lipid deposition, and alter protein acetylation. Mitochondrial dysfunction and neuroinflammation are thought to synergistically activate a cycle of deleterious events leading to neuronal death [39]. Microglia, thought to be primary mediators of neuroinflammation, release potentially harmful factors, including reactive oxygen species and pro-inflammatory cytokines that can damage mitochondria [40]. It is known that neural mechanisms are involved in inflammatory responses in brain [41] and that the outcome of certain neurodegenerative disorders is influenced by the balance between pro- and anti-inflammatory mediators [42]. The results showing the ability of acetate to completely attenuate microglia activation and IL-1β level further supports these findings. Mitochondrial disruption in microglia cell culture stimulated with LPS disrupts the balance between pro- and anti-inflammatory cytokine production, suggesting that mitochondrial integrity is necessary to support injury resolution [43]. In this regard, acetate treatment in cultured microglia increases the transcription of the anti-inflammatory cytokines transforming growth factor-beta 1 (TGF-β1) and IL-4, suggesting that acetate-induced histone modulation may influence more strongly the expression of anti-inflammatory cytokines [16]. However, acetate also reduces the expression of pro-inflammatory cytokines Il-1β and TNFα in microglia without altering RNA. Thus while an increase in the expression of anti-inflammatory cytokines may have a role in reducing microglial activation, other alterations in inflammatory signaling that are influenced by protein acetylation are also likely involved. Furthermore, primary mitochondrial dysfunction resulting from exposure to neurotoxins induces microglial activation [39] of which can trigger neurodegeneration [40,44]. Taken together, these data provide a reasonable rationale to suggest that stimulating acetyl-CoA metabolism in rats subjected to *B. burgdorferi* may influence neuroglial communication and possibly disrupt the progression of Lyme neuroborreliosis.

## Conclusions

Rats inoculated with *B. burgdorferi* demonstrate characteristics of microglia activation that is reversed to control levels when treated prophylactically with acetate supplementation. In this regard, acetate supplementation was able to completely resolve morphological changes in resident microglia, attenuated CD11b to control levels, and reduced brain-derived IL-1β levels as compared to rats that were inoculated with *B. burgdorferi*. Interestingly, infected rats did not demonstrate reactive astrogliosis as characteristic in primate models of Lyme neuroborreliosis. This, we believe, is a result of a significantly lower bacterial challenge in our study as compared to primate models, where considerably more bacteria are directly introduced into the brain. Further, these results support the premise that acetate supplementation is effective at reducing TLR2-mediated neuroinflammation that may be attributed to stimulating brain energy supplementation or a disruption of microglia-derived pro-inflammatory gene expression.

## Abbreviations

Acetyl-CoA: acetyl coenzyme A; BSA: bovine serum albumin; CD11b: integrin alpha M chain; CSF: cerebrospinal fluid; ELISA: enzyme-linked immunosorbent assay; GFAP: glial fibrillary acidic protein; GTA: glyceryl triacetate; Ig: immunoglobulin; IL-1β: interleukin-1 beta; LPS: lipopolysaccharide; PBS: phosphate-buffered saline; qPCR: quantitative polymerase chain reaction; TGF-β1: transforming growth factor-beta 1; TLR: toll-like receptor; TNFα: tumor necrosis factor alpha.

## Competing interests

The authors declare they have no competing interests.

## Authors’ contributions

CAB and TAR participated in the research design of the experiments outlined in this study. The experiments and data analysis were performed by CAB, AMF, HMH, and TAR. In addition, CAB and TAR wrote or contributed to the writing of the manuscript. All authors have read and approved the final version of the manuscript.
